# Alcohol consumption exacerbates high-fat diet-mediated disruptions in myelopoiesis and osteoclastogenesis in mouse models of metabolic dysfunction-associated liver diseases

**DOI:** 10.3389/fendo.2026.1783132

**Published:** 2026-03-20

**Authors:** Hami Hemati, Madison B. Blanton, Lauren Rutt, Nicholas Keiran, Rebecca Geron, Florence Lima, Rebecca L. McCullough, Ilhem Messaoudi

**Affiliations:** 1 Microbiology, Immunology and Molecular Genetics, College of Medicine, University of Kentucky, Lexington, KY, United States; 2 Pharmaceutical Sciences, College of Pharmacy, University of Kentucky, Lexington, KY, United States; 3 Department of Pharmaceutical Sciences, Skaggs School of Pharmacy and Pharmaceutical Sciences, University of Colorado Anschutz Medical Campus, Aurora, CO, United States; 4 Nephrology, Bone and Mineral Metabolism, College of Medicine, University of Kentucky, Lexington, KY, United States; 5 Alcohol Research Program, University of Colorado Anschutz Medical Campus, Aurora, CO, United States

**Keywords:** alcohol consumption, hematopoietic stem and progenitor cells, high-fat diet, MASLD, metabolic dysfunction-associated liver diseases, MetALD, osteoclast, osteoclastogenesis

## Abstract

**Objective:**

Metabolic Dysfunction-Associated Steatotic Liver Disease (MASLD) and Metabolic Dysfunction-Associated Alcohol-related Liver Disease (MetALD) exhibit systemic immune abnormalities. Given that such immune dysregulation is closely linked to the skeletal complications frequently observed in MASLD and MetALD, we sought to comprehensively characterize the bone marrow hematopoietic compartment and its link to osteoclastogenesis.

**Methods:**

We utilized bone marrow from mouse models of MASLD (high-fat diet, HFD) and MetALD (high-fat diet plus ethanol, HFD+EtOH), followed by flow cytometric analysis to phenotype hematopoietic stem and progenitor cells (HSPCs), as well as *in vitro* and *in vivo* assays to evaluate osteoclastogenesis.

**Results:**

We found that HFD depletes the hematopoietic stem cell (HSC) and early multipotent progenitors, whereas HFD+EtOH preserves HSC and skews hematopoiesis toward myeloid-committed multipotent progenitors, resulting in the expansion of Ly6Chigh progenitors and monocytes. Further, enrichment of committed osteoclast precursors (CD115^+^RANK^+^) was significantly greater in the HFD+EtOH compared to HFD alone. This was associated with increased RANK expression in Ly6Chigh precursors and was accompanied by enhanced osteoclast multinucleation, cell area, and elevated resorptive activity in osteoclasts derived from the HFD+EtOH group. Bone analysis revealed elevated osteoclast activity, along with increased epiphyseal area and femur length, in HFD+EtOH-fed mice, potentially due to delayed chondrocyte hypertrophy/prolonged growth plate activity.

**Conclusion:**

Collectively, these findings demonstrate that while both HFD and HFD+EtOH trigger diet-specific hematopoietic alterations, alcohol amplifies the HFD-induced effects, promoting the expansion of myeloid and osteoclast precursors, thereby enhancing osteoclastogenesis. These findings highlight the need to consider hematopoietic health in patients with MASLD and MetALD and lay the groundwork for future research focused on alleviating immune and skeletal complications associated with metabolic dysfunction-related liver diseases.

## Introduction

Alcohol misuse and obesity are leading and increasingly intersecting drivers of global morbidity and premature mortality, acting in large part through profound disruption of immune and metabolic homeostasis that promotes progressive liver injury (1, 2). These overlapping insults underpin the spectrum of metabolic dysfunction-associated liver diseases. Patients with hepatic steatosis and at least one cardiometabolic risk factor but without significant alcohol intake are classified as having metabolic dysfunction-associated steatotic liver disease (MASLD), formerly termed non-alcoholic fatty liver disease (NAFLD) (3–5). With an estimated global prevalence of about 30% and rising alongside the obesity epidemic, MASLD has become a major public health concern (3–5). In contrast, those who meet the same criteria but also consume moderate to high levels of alcohol (6) (typically 20–50 g/day for women and 30–60 g/day for men, or weekly 140–350 g for women and 210–420 g for men) are classified as having metabolic dysfunction-associated alcohol-related liver disease (MetALD) (7). While the prevalence of MetALD is lower (2.2%), patients with MetALD may experience faster progression of liver disease, underscoring the need for separate investigation and tailored management strategies (7).

MASLD and MetALD are associated with local and systemic immune dysregulation (5, 8), which play crucial roles in disease progression and extrahepatic manifestations (9, 10). Chronic alcohol consumption and its associated liver injury also disrupt hematopoiesis through mechanisms such as ethanol toxicity, oxidative stress, and dysregulation of cytokine and chemokine networks, impairing the bone marrow’s capacity to sustain normal blood cell production (4, 11). This highlights a reciprocal relationship in which the liver’s metabolic state profoundly shapes hematopoiesis, while hematological dysfunction can, in turn, exacerbate liver pathology. In fact, a large National Health and Nutrition Examination Survey (NHANES)-based study (n = 10,308 MASLD patients) reported that elevated monocyte and neutrophil counts, monocyte-to-lymphocyte ratio (MLR), neutrophil-to-lymphocyte ratio (NLR), and platelet-to-lymphocyte ratio (PLR), and lower levels of lymphocytes and platelets, were significantly associated with increased all-cause mortality in MASLD (12). These markers reflect systemic inflammation and immune activation, suggesting that MASLD patients experience a shift toward myeloid dominance. While specific large-scale data on MetALD is limited, as it represents a synergistic pathology combining MASLD and moderate to high alcohol consumption, extrapolations from alcohol-related liver disease suggest neutrophilia and monocytosis are common, especially during active inflammation or alcoholic hepatitis (13). Despite these insights, the molecular mechanisms by which MASLD and MetALD reshape lineage commitment in the bone marrow remain unclear.

The crosstalk between the immune system and bone, mediated by factors such as RANKL, TNF-α, and IL-6, suggests that MASLD and MetALD may actively contribute to skeletal deterioration (14). In fact, skeletal complications in these conditions are increasingly recognized and span both bone and muscle disorders (15–19). In MASLD, impaired osteoblast and osteoclast function and reduced bone mineral density (BMD) are strongly associated with disease burden (17, 18, 20, 21). Although direct evidence for MetALD-related bone disease is limited, alcohol can independently disrupt bone remodeling. When combined with MASLD-associated inflammation, MetALD likely accelerates skeletal complications, with heightened risk observed in post-transplant settings (22).

Overall, high-fat diet and alcohol consumption, separately and together, modulate the crosstalk between bone marrow hematopoiesis and bone tissue homeostasis in complex ways. Understanding this relationship is crucial for developing comprehensive treatment strategies that address both hepatic and hematopoietic dysfunctions. Yet, most prior studies on MASLD and MetALD have primarily focused on liver-resident or circulating immune cells (4, 23). In this study, we employed HFD and HFD+EtOH mouse models to mimic MASLD and MetALD, respectively, and examined alterations in HSPCs that may underlie the subsequent changes observed in bone marrow and bone. Our findings highlight the need to consider hematopoietic health in patients with metabolic and alcohol-related liver disease, paving the way for novel treatment strategies for immune-skeletal complications.

## Materials and methods

### Mouse models

Male wildtype C57BL/6 mice (5-weeks old) were housed two per cage and randomly assigned to one of three groups: chow (n=10), high-fat diet (HFD; n=10), or HFD combined with ethanol (HFD+EtOH; n=12). Animals were age- and weight-matched before group assignment. For 12 weeks, a high fructose, high fat, high cholesterol diet (“GAN” diet, 40% kcal fat, 20% kcal fructose, 2% cholesterol; D09100310i, Research Diets, Inc.) was available *ad libitum* to HFD and HFD+EtOH groups, and a chow diet consisting of 4.5% fat was available *ad libitum* to controls. During week 8, the ethanol group received thrice weekly oral gavages of ethanol (4 g/kg), followed by thrice weekly gavages of ethanol (5g/kg) on weeks 9-12. During weeks 8-12, the HFD group was administered 0.9% sterile saline by gavage, while the control group received water gavages with identical timing and frequency. All gavages were administered between 8–10 am. Mice were sacrificed 6 hours after the final gavage on week 12. Mice were anesthetized with a ketamine/xylazine/acepromazine cocktail (100 mg/mL each), administered intraperitoneally at 0.1 mL/25 g body weight. Euthanasia was performed by exsanguination under deep anesthesia. To harvest bones, hind limbs were removed at the pelvic joint under sterile conditions, and intact femur–tibia units were collected and stored in 15 mL conical tubes containing chilled RPMI 1640 (Gibco) supplemented with 2% fetal bovine serum (FBS, Corning) and penicillin-streptomycin (Gibco), sealed with parafilm, and shipped overnight on ice packs. The animal model was approved by the University of Colorado Anschutz Medical Campus Institutional Animal Care and Use Committee.

### Bone marrow collection

Bones were shipped overnight at 4 °C and processed within 24 hours of harvest. Briefly, residual muscle, tendons, and skin were carefully removed, and femurs and tibias were separated at the knee joint. The epiphyses were excised, and bone marrow was flushed from the medullary cavity with phosphate-buffered saline (PBS) with a 1 mL syringe fitted with a 25G needle. The resulting cell suspension was centrifuged at 2300 rpm for 15 minutes. Cell pellets were resuspended and subjected to red blood cell lysis, followed by two washes with PBS. The recovered mononuclear cells were counted and subsequently prepared for downstream applications (24).

### Spectral flow cytometry and dimensionality reduction analysis

Bone marrow cells were incubated with TruStain FcX Fc Receptor Blocking Solution (Biolegend) for 10 mins at 4 °C. Samples were washed with PBS and then stained for 30 minutes at 4 °C with cell surface antibodies; B220 (CD45R; PE-Cyanine7 or PerCP), CD3 (PE-Cyanine7 or PerCP), TER-119 (PE-Cyanine7 or PerCP), CD11b (Brilliant Violet 650 or PerCP/Cyanine5.5), Ly-6C (PE or Brilliant Violet 605), Ly-6G (Brilliant Violet 510), CD34 (FITC or APC), CD16/CD32 (PerCP-Cyanine5.5 or Brilliant Violet 711), Ly-6A/E (Sca-1; PB or PE), c-Kit (CD117; APC-Cyanine7), CD48 (FITC), CD150 (SLAM; Brilliant Violet 421), CD115 (CSF1R, Brilliant Violet 711 or PE-eFluor610), CD135 (Alexa Fluor 700), and RANK (CD265; Alexa Fluor 647) in the presence of True-Stain Monocyte Blocker (Biolegend) and Brilliant Stain Buffer (BD Biosciences). The samples were then washed with PBS and acquired using an Attune NxT Flow Cytometer (Thermo Fisher Scientific). Data were analyzed using FlowJo v10.10. Acquired events were cleaned using FlowAI (v2.3.22) (25). Dimensionality reduction was carried out with EmbedSOM (v2.2.0) (26). Cell clusters were identified using either FlowSOM (v4.1.04) (27) or Phenograph (v2.5.05) (28). To visualize relationships among clusters, PHATE (Potential of Heat-diffusion for Affinity-based Trajectory Embedding; v1.0.0) (29) was applied.

### *In vitro* osteoclastogenesis assay and TRAP staining

The bone marrow mononuclear cells were seeded overnight at 37 °C (5% CO_2_) in a 48-well plate containing MEMα with GlutaMAX™ Supplement (Gibco), 10% FBS, 1% penicillin-streptomycin, and 10 ng/mL mouse M-CSF (Peprotech) at a density of 0.5×10^6^ cells/well. The medium was collected and centrifuged to obtain the non-adherent cells. Cells were then incubated in induction medium (MEMα, 10% FBS, 1% penicillin-streptomycin, 30 ng/mL mouse M-CSF, and 50 ng/mL mouse RANKL) for 6 days, with the induction medium replaced every 48 hours. The culture supernatant was discarded 24 hours following the third media change. The cells were then processed for a Tartrate-resistant acid phosphatase (TRAP) staining using a kit following the manufacturer’s recommendations (Takara Bio), as previously described (30). Briefly, cells were fixed with Fixation base solution for 5 minutes, then washed with sterile distilled water and incubated with TRAP solution at 37 °C for 40 minutes. The substrate was removed, and the plates were washed three times. The plates were imaged on Cytation 5 (Agilent). The images were analyzed using ImageJ software, and TRAP-positive cells with three or more nuclei were considered osteoclasts.

### Bone resorption assay

Bone resorption capacity was determined using a bone resorption assay kit with fluoresceinated calcium phosphate-coated plate (Cosmo Bio) according to the manufacturer’s instructions, as previously described (30). Briefly, the calcium phosphate-coated plates were incubated at 37 °C for 2 hours in the dark with fluoresceinamine-labeled chondroitin sulfate and then washed twice with PBS. The non-adherent bone marrow cells were obtained as described above. The wells were then seeded at a density of 0.2 × 10^6^ cells/well in MEMα, supplemented with 10% FBS, 1% penicillin-streptomycin, 1% GlutaMax (Thermo Fisher), 30 ng/ml mouse M-CSF, and 100 ng/ml mouse RANKL. The media was collected and refreshed every 48 hours. After 6 days, cells were removed by 5% sodium hypochlorite. Plates were then washed with water and stored at 4 °C until imaging. Pits representing resorbed areas were analyzed using ImageJ.

### Bone decalcification

The femurs were fixed with 10% neutral buffered formalin for 24 hours. The bones were then washed and incubated in 20% Ethylenediaminetetraacetic acid (EDTA; pH 7.4), refreshed every 3 days for ~2 weeks at 4 °C with shaking, until they became soft and pliable. The bones were then rinsed with water, merged in 30% sucrose, and submitted for sectioning to the University of Kentucky’s Biospecimen Procurement and Translational Pathology Shared Resource.

### Histological analyses

Decalcified bones were processed with an Excelsior AS tissue processor (Epredia) and embedded in paraffin. Sections of 4 µm thickness were cut using a manual microtome and mounted on Flex Plus slides (Dako). Slides were dried upright at 58 °C for at least 1 hour before hematoxylin and eosin (H&E) staining was performed on a Tissue-Tek Prisma stainer, followed by mounting with Cytoseal permanent mounting medium (Richard Allen). The growth plate (epiphyseal plate), epiphyseal area, and epiphyseal height were quantified on H&E-stained sections of the distal femur. Total femur length was measured from the proximal to the distal end. All measurements were performed using QuPath software (v0.6.0). In all measurements, the investigator was blinded to experimental group identity.

### Trichrome and TRAP staining

Formalin-Fixed Paraffin-Embedded (FFPE) bone sections were placed on positively charged slides and baked at 58 °C for at least 1 hour. Slides were then deparaffinized and rehydrated through a graded series of solutions, followed by trichrome staining using the MasterTech One-Step Trichrome Kit (Statlab KTTRGPT) according to the manufacturer’s instructions, with the modification of using Weigert’s hematoxylin for nuclear staining. Positive control tissue was included to verify assay performance. The TRAP staining was performed on deparaffinized and rehydrated tissues as described above for *in vitro* differentiated osteoclasts. The TRAP-positive areas were quantified in matching distal regions using pixel classification in QuPath software.

### Statistical analyses

Outliers were identified using ROUT analysis at a Q-value of 0.1% and removed from the dataset. We then performed the Shapiro-Wilk test (α = 0.05) to determine if the Gaussian requirements were met. Normally distributed data were analyzed for significance via a paired or unpaired t-test, with Welch’s correction (parametric) or Mann-Whitney (non-parametric). Comparisons between three or more groups were conducted using a one-way ANOVA with the Holm-Sidak test to adjust for multiple comparisons (confidence level set at 0.05). A p-value of ≤0.05 was considered statistically significant, and a p-value 0.1≥X≥0.05 was denoted as a modest change.

## Results

### HFD and HFD+EtOH are associated with different outcomes for the HSCs pool

**Figure 1:**
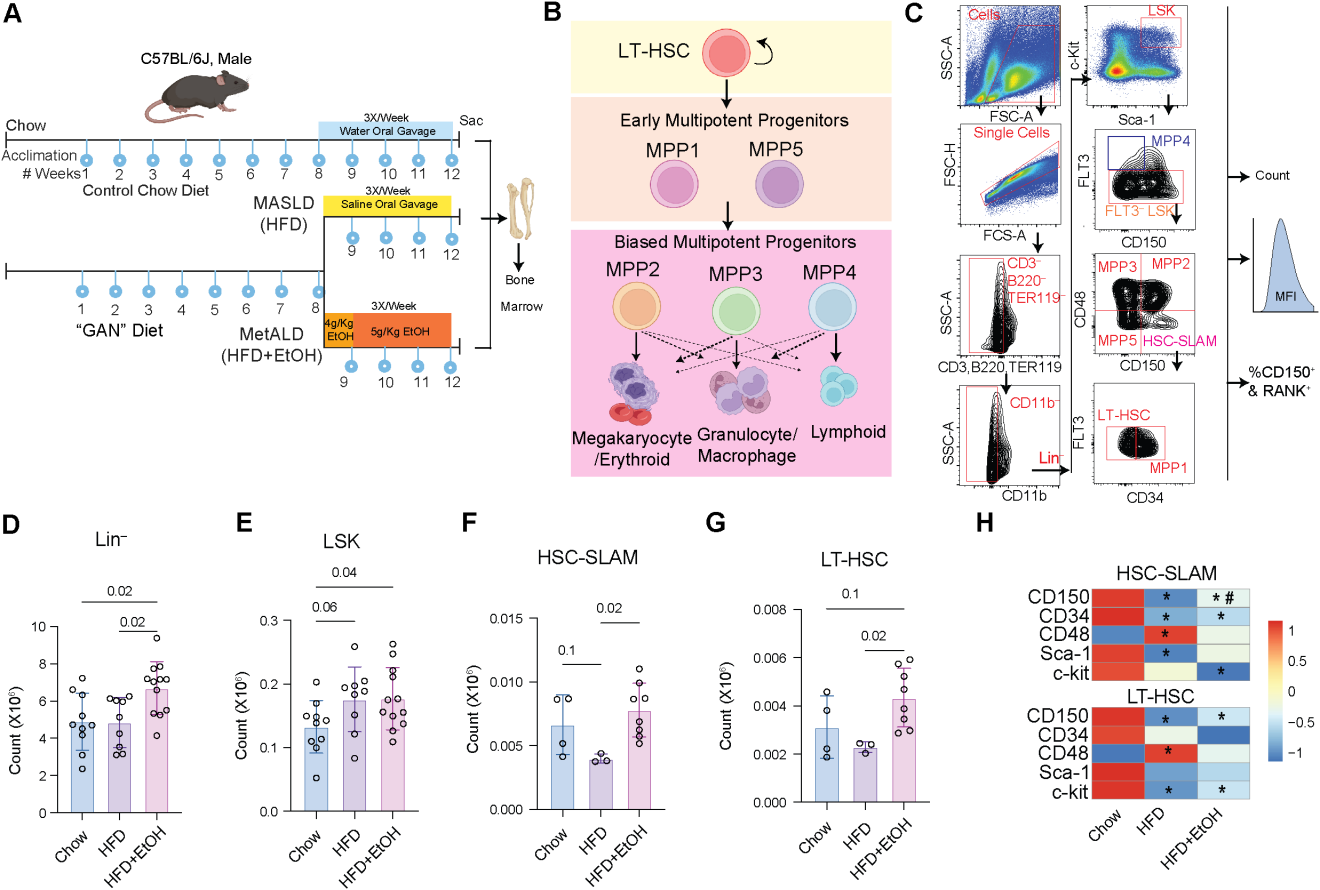
HSC pool was increased after the HFD+EtOH diet. **A**) Study Design**. B**) Differentiation of HSCs to early and committed MPPs. **C**) Gating Strategy used to phenotype bone marrow HSPCs. Number of **D**) Lin^–^, **E**) LSK cells, **F**) HSC-SLAM, and **G**) LT-HSCs. **H**) MFI of key hematopoietic markers on the surface of HSCs. The MFIs were normalized across the groups. * in the HDF box, indicates a p-value ≤0.05 between Chow and HFD. * in the HFD+EtOH box indicates a p-value ≤0.05 between Chow and HFD+EtOH. # in the HFD+EtOH box indicates a p-value ≤0.05 between HFD+EtOH and HFD.

To assess and compare the impact of HFD and HFD+EtOH on hematopoiesis, the bone marrow was harvested after 12 weeks (**Figure 1A**). In mouse models, bone marrow-resident long-term hematopoietic stem cells (LT-HSCs), while possessing self-renewal capacity, differentiate into early multipotent progenitors 1 and 5 (MPP1 and MPP5), which further give rise to biased progenitor cells, including myeloid-biased early multipotent progenitors 2 and 3 (MPP2 and MPP3), as well as lymphoid-biased early multipotent progenitors 4 (MPP4) (31–33) (**Figure 1B**). These hematopoietic stem and progenitor cells (HSPCs) were identified using flow cytometry, after which their absolute counts and the median fluorescence intensity (MFI) of markers within each population were determined (**Figure 1C**).

The HFD+EtOH diet resulted in a significant expansion of lineage-negative (Lin^–^; CD3^–^Ter119^–^B220^–^CD11b^–^) cells and LSKs (Lin^–^, Stem cell antigen 1 (Sca1) positive, c-Kit (CD117) positive; Lin^–^Sca1^+^c-Kit^+^), accompanied by a modest increase in FLT3^–^ LSK subset compared to chow (**Figures 1D–G**; **Supplementary Figures 1A, B**). Additionally, relative to HFD, the HFD+EtOH significantly increased the number of Lin^–^ cells, HSC-SLAM (HSC-Signaling Lymphocytic Activation Molecule; CD150^+^CD48^–^) (34), and long-term (LT)-HSCs (**Figures 1D–G**; **Supplementary Figures 1A, B**). In contrast, HFD alone induced a modest reduction in HSC-SLAM cells compared with chow, consistent with the notable decrease in their frequency among FLT3^–^ LSKs, ultimately leading to a significant loss of LT-HSCs (**Figures 1D–G**; **Supplementary Figures 1A, B**). These findings suggest that HFD alone leads to attrition of the HSC pool, whereas HFD+EtOH, while significantly expanding lineage-negative cells, also expands the HSC pool.

Assessment of key surface markers on HSCs revealed downregulation of CD150 (SLAMF1; signaling lymphocytic activation molecule family member 1) in both HFD and HFD+EtOH HSCs. Lower CD150 expression on HSCs is correlated with a more robust differentiation capacity towards lymphoid cells (35–37). CD34 was also downregulated on HSC-SLAM in both experimental groups, while Sca-1 was only downregulated in HSC-SLAM from the HFD group (**Figure 1H**; **Supplementary Figure 1C**). These changes are important as lower CD34 and c-kit indicate a shift towards a self-renewing state (38–40), while Sca-1 downregulation is associated with reduced self-renewal capacity (41). Therefore, the downregulation of Sca-1 and comparable levels of c-kit expression on HSC-SLAMs between the HFD and chow groups provide a potential explanation for the reduced self-renewing capacity of HSCs with HFD, potentially accounting for the depletion of these cells.

These findings suggest that these diets differentially impact hematopoietic stem cells. HFD+EtOH may enhance the self-renewing capacity of HSCs, enabling them to maintain their numbers despite increased differentiation into lineage-negative cells. In contrast, HFD alone appears to be associated with enhanced HSC differentiation while impairing their self-renewal, resulting in the depletion of the HSC pool.

### Alcohol combined with HFD is associated with enhanced myeloid-biased multipotent progenitors

**Figure 2:**
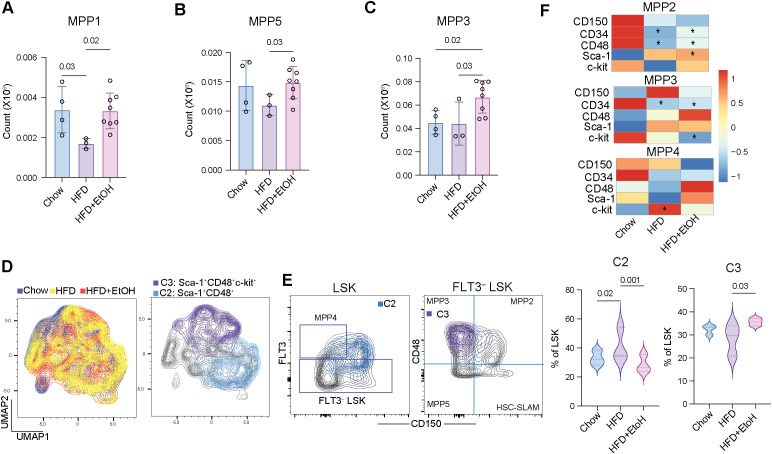
Alcohol in combination with HFD enhances myeloid cell commitment. Cell count of **A**) MPP1, **B**) MPP5, and **C**) MPP3 cells. **D**) UMAP (Uniform Manifold Approximation and Projection) plots illustrating the distribution of groups (left) and C2 and C3 clusters (right). **E**) Overlapping C2 and C3 clusters on supervised gating plots of LSKs and FLT3^–^ LSKs. **F**) MFI of key hematopoietic markers on the surface of committed multipotent progenitors MPP2, MPP3, and MPP4. * in the HDF box, indicates a p-value ≤0.05 between Chow and HFD. * in the HFD+EtOH box indicates a p-value ≤0.05 between Chow and HFD+EtOH.

We further assessed the changes in early and committed multipotent progenitors. Consistent with observations for HSCs, a significant depletion of early progenitors MPP1 (Lin^–^Sca-1^+^ckit^+^FLT3^–^CD48^–^CD150^+^CD34^+^) was observed in HFD compared to chow and HFD+EtOH (**Figure 2A**). Early progenitors MPP5 (Lin^–^Sca-1^+^ckit^+^FLT3^–^CD48^–^CD150^–^) were also decreased in HFD compared to HFD+EtOH (**Figure 2B**). Myeloid-biased MPP3 (Lin^–^Sca-1^+^ckit^+^FLT3^–^CD48^+^CD150^–^) were, however, significantly increased in HFD+EtOH compared to both chow and HFD (**Figure 2C**). Yet, there were no significant differences in the number of MPP2 (Lin^–^Sca-1^+^ckit^+^FLT3^–^CD48^+^CD150^+^) or MPP4 (Lin^–^Sca-1^+^ckit^+^FLT3^+^CD150^–^) between the groups (**Supplementary Figure 1D**). To detect populations that could be overlooked in supervised analyses, we conducted unsupervised FlowSOM clustering on LSKs. Among the 8 identified clusters, C2 (Sca-1^+^CD48+) was enriched in the HFD compared to the other two groups. Overlay of the C2 cluster with conventionally gated populations revealed that this cluster may correspond to a transient hematopoietic progenitor population (42). Finally, the C3 cluster (a Sca-1^+^CD48^+^c-kit^+^ subset of MPP3) was enriched in HFD+EtOH compared to the HFD (**Figures 2D, E**; **Supplementary Figure 1E**).

Furthermore, CD34 expression was reduced in MPP2, MPP3, and MPP5 under both experimental diets (**Figure 2F**; **Supplementary Figures 1F, G**). CD34 is commonly expressed on early progenitor cells; its downregulation signals a more restricted differentiation potential, with a specific push toward mature myeloid cells and megakaryocytes (31, 32, 43). Sca-1 was, however, upregulated in MPP2 cells of the HFD+EtOH group (**Figure 2F**). Sca-1 usually does not show significant expression in megakaryocyte/erythroid progenitors; however, its enhanced expression indicates stress conditions and signals the activation of emergency or extramedullary hematopoiesis to boost rapid platelet/red cell production (44). c-Kit was upregulated on MPP4s in the HFD group but downregulated on MPP1, MPP5, and MPP3 in the HFD+EtOH group (**Figure 2F**; **Supplementary Figure 1G**). While upregulation of c-Kit is typically associated with maintenance of an early proliferative state, its expression gradually decreases with terminal lineage commitment (45, 46). Therefore, the downregulation of c-Kit may indicate that early and committed progenitors are more actively differentiating into late-stage granulocytic or monocytic lineages.

Collectively, these data indicate that HFD primarily impacts hematopoiesis by depleting the HSC pool, which consequently reduces the number of early multipotent progenitors. In contrast, HFD+EtOH was associated with a shift toward myeloid lineage differentiation.

### HFD+EtOH skews hematopoiesis toward monocyte progenitors

**Figure 3:**
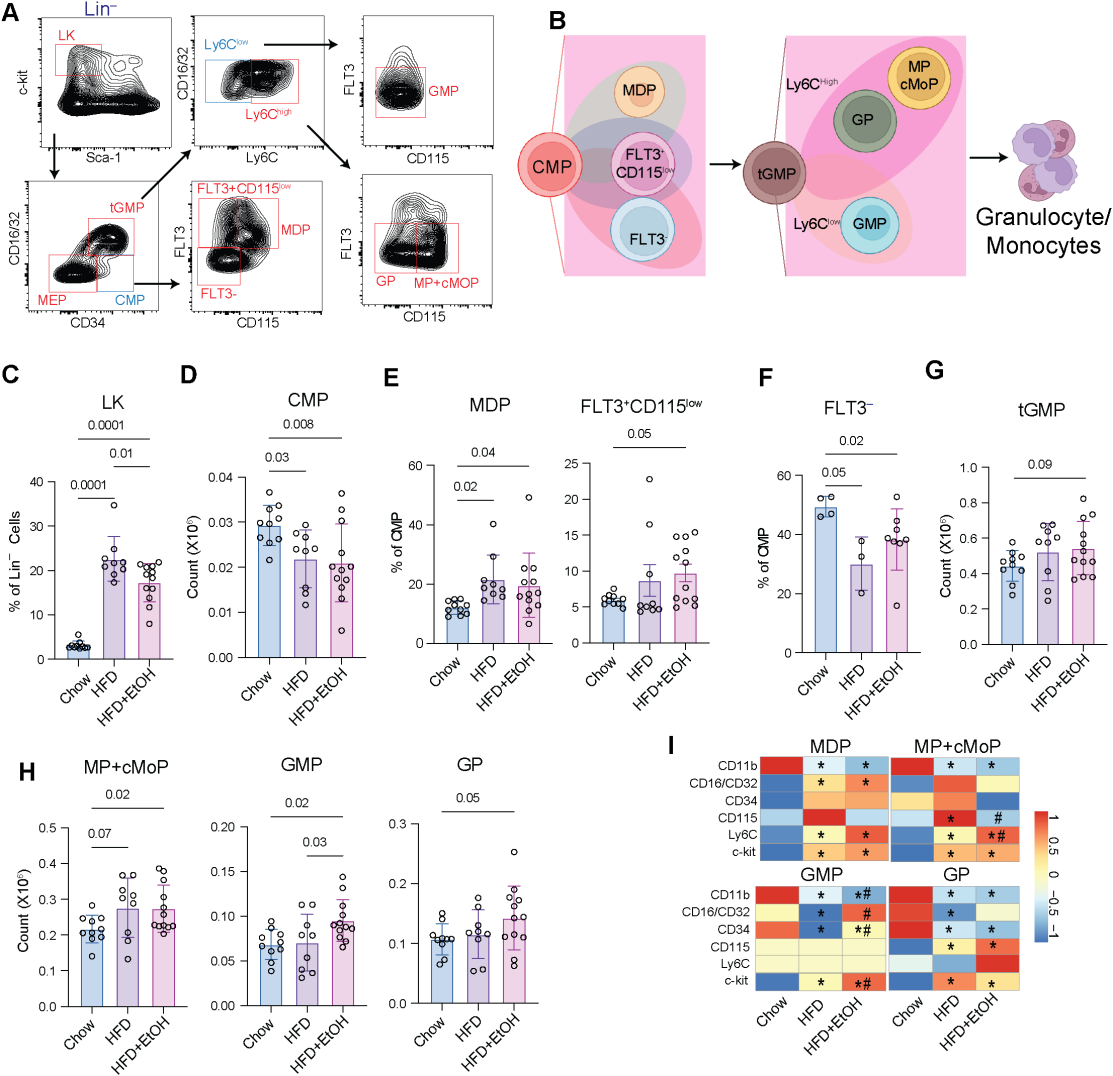
HFD+EtOH skews hematopoiesis toward granulocyte-monocyte progenitors. **A)** Gating strategy used to assess subsets of LK cells. **B)** The differentiation trajectory and subsets of CMP and tGMPs. **C)** The frequency of LK cells among lineage-negative cells. **D)** The number of CMP cells. The frequency of **E)** MDP, FLT3^+^CD115^low^, and **F)** Flt3^–^ subsets within the CMP population. The number of **G)** tGMP**, H)** MP+cMoP, GMP, and GP populations**. I)** The MFI of cell surface markers. The MFIs were normalized across the groups. * in the HDF box, indicates a p-value ≤0.05 between Chow and HFD. * in the HFD+EtOH box indicates a p-value ≤0.05 between Chow and HFD+EtOH. # in the HFD+EtOH box indicates a p-value ≤0.05 between HFD+EtOH and HFD.

Given the shift towards myeloid progenitors with HFD+EtOH, we next assessed granulocyte and monocyte progenitor populations among LK (Lin^–^c-Kit^+^Sca-1^–^) cells using flow cytometry (**Figures 3A, B**). Both diets resulted in a significantly increased proportion of LK cells within the Lin^–^ compartment compared to chow (**Figure 3C**), further indicating enhanced differentiation of multipotent progenitors toward lineage-committed progenitors. Among these progenitors, the MEPs (megakaryocytic-erythroid progenitor; Lin^–^c-kit^+^Sca1^–^CD16/32^–^CD34^–^) showed only a modest increase in HFD+EtOH relative to the chow (**Supplementary Figure 1H**).

However, the number of common myeloid progenitors (CMPs; Lin^–^c-kit^+^Sca1^–^CD16/32^–^CD34^+^) in the HFD and HFD+EtOH were significantly decreased compared to the chow (**Figure 3D**). To determine whether this resulted from enhanced differentiation of CMPs into granulocyte or monocyte progenitor cells, we assessed the absolute numbers and the frequencies of CMP subsets based on expression of FLT3 and CD115. Within CMPs, the frequency of MDPs (monocyte-dendritic cell progenitors; FLT3^+^CD115^+^) was significantly elevated in both experimental groups compared to the chow; whereas FLT3^+^CD115^low^, a DC-biased subset (47–49), was only significantly increased in HFD+EtOH compared to chow (**Figure 3E**). Absolute numbers of these two subsets were, however, unchanged (**Supplementary Figure 1I**), suggesting relative enrichment of these subsets within the parent CMP pool rather than their absolute expansion. By contrast, the FLT3^–^ subset, as an early granulocyte-monocyte progenitor (GMPs) (49), was significantly decreased both proportionally and in absolute numbers following both diets (**Figure 3F**; **Supplementary Figure 1J**).

To assess whether the FLT3^–^ subset, which accounts for more than 50% of CMPs in chow, is decreased due to enhanced differentiation toward GMPs, we analyzed granulocyte-monocyte progenitors. The total GMPs (tGMP: Lin^–^c-kit^+^Sca1^–^CD16/32^+^CD34^+^) showed a modest increase in HFD+EtOH compared to chow (**Figure 3G**). We used expression level of Ly6C, FLT3 and CD115 to sub-divide tGMP (49) into MP+cMoP (monocyte progenitors + common monocyte progenitors; Lin^–^c-kit^+^Sca1^–^CD16/32^+^CD34^+^Ly6C^high^FLT3^–^CD115^+^), GP (granulocyte progenitors; Lin^–^c-kit^+^Sca1^–^CD16/32^+^CD34^+^Ly6C^high^FLT3^–^CD115^–^), and GMP (Lin^–^c-kit^+^Sca1^–^CD16/32^+^CD34^+^Ly6C^low^FLT3^–^CD115^low^). All three populations were significantly enriched in HFD+EtOH compared to the chow diet (**Figure 3H**).

We next examined the expression of key cell surface markers on these progenitors (**Figure 3I**; **Supplementary Figure 1K**). While c-kit was upregulated, CD11b was downregulated in all monocyte and granulocyte progenitors (MDP, MP+cMoP, GMP, GP). As HSPCs mature toward the myeloid lineage, they gradually lose c-Kit expression and acquire CD11b (46). Therefore, enhanced c-Kit expression accompanied by reduced CD11b suggests a block or delay in terminal myeloid maturation, maintaining these cells in a more immature, progenitor-like state. We also observed enhanced expression of CD16/CD32 on GMPs in the HFD+EtOH group, whereas it was downregulated in HFD alone. This suggests that GMPs in the HFD+EtOH group are primed for rapid differentiation along granulocytic and monocytic pathways (46, 50). Furthermore, the downregulation of CD34 in GMPs and GPs indicates a more restricted differentiation potential of these cells (46, 51). Finally, an elevated Ly6C expression on MDP and MP+cMoP cells was found in both the HFD and HFD+EtOH groups compared to the chow group (**Figure 3I**), supporting an increased potential for monocyte differentiation. Enhanced Ly6C expression on monocyte progenitors marks a strong monocyte-committed fate, with a high ability to differentiate into classical Ly6C^high^ monocytes, which are the main inflammatory monocyte subset in mice (49, 52, 53).

Together, these findings support the notion that the reduction in total CMPs is due to their enhanced differentiation into granulocytic and monocytic progenitors, particularly following the HFD+EtOH diet. The observed upregulation of c-Kit and CD16/CD32, along with elevated Ly6C expression, indicates that these progenitor populations are potentially more proliferative, lineage-primed, and committed towards inflammatory myeloid cells.

### HFD+EtOH increases bone marrow inflammatory Ly6C^high^ monocytes

**Figure 4:**
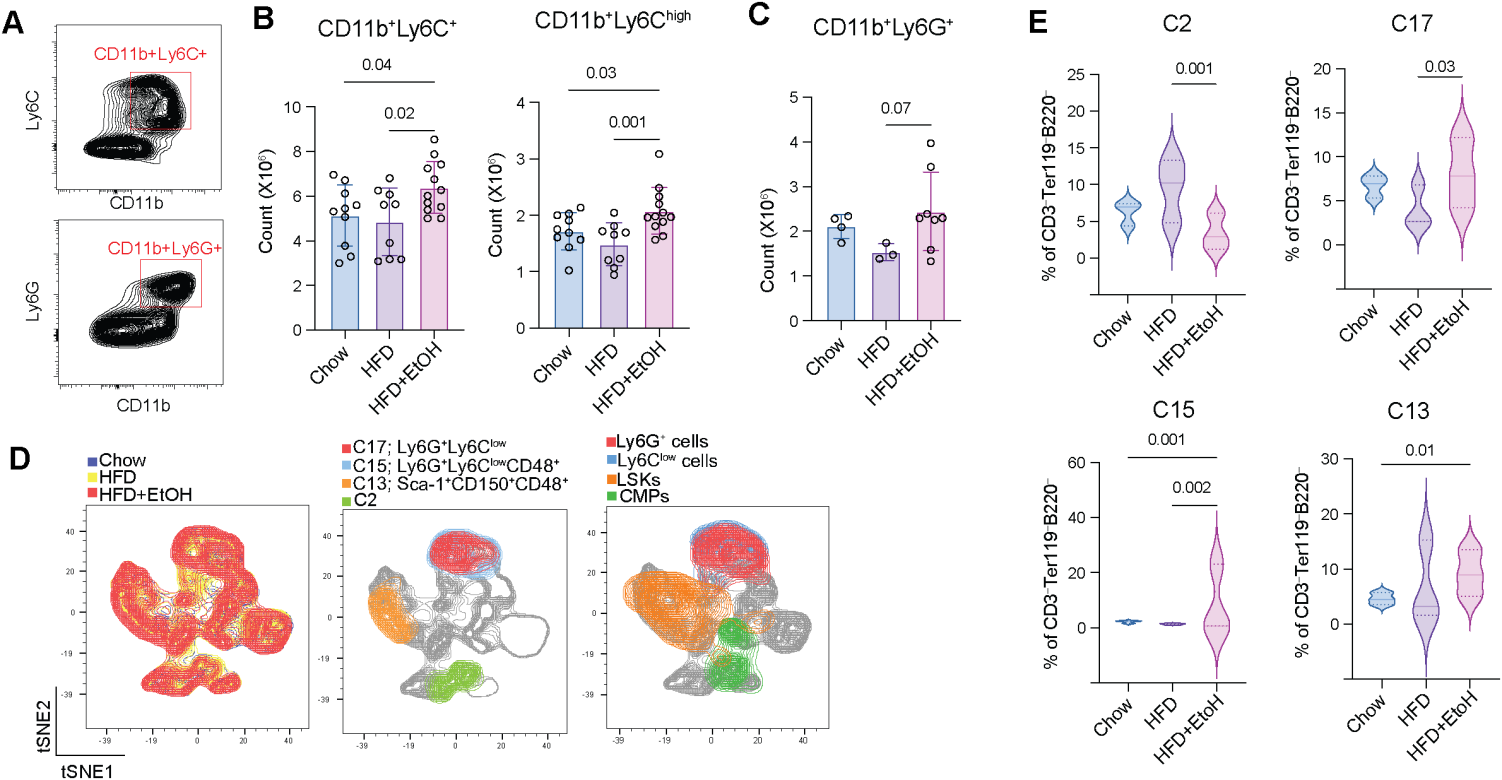
HFD+EtOH skews hematopoiesis toward monocyte production. **A)** Gating strategy to detect CD11b^+^Ly6C^+^ and CD11bLy6G^+^ cells. **B**) The number of CD11b^+^Ly6C^+^ and CD11b^+^Ly6C^high^ and **C**) CD11b^+^Ly6G^+^ cells. **D)** t-SNE (t-distributed Stochastic Neighbor Embedding) plots illustrating groups and identified clusters that were significantly different among the groups. Supervised gated populations were overlapped on plots. **E)** The frequency of clusters among the total CD3^–^B220^–^Ter119^–^ cells.

We further quantified the mature monocytes and neutrophils among total bone marrow cells to assess the differentiation potential of granulocyte and monocyte progenitors (**Figure 4A**). There was a significant increase in the absolute count of CD11b^+^Ly6C^+^ and potential inflammatory CD11b^+^Ly6C^high^ monocytes (52, 53) in HFD+EtOH compared to both HFD and chow (**Figure 4B**) accompanied by enhanced Ly6C expression on monocyte progenitor cells (**Figure 3I**). CD11b^+^Ly6G^+^ neutrophils were, however, only modestly increased in HFD+EtOH compared to HFD (**Figure 4C**).

We performed an unsupervised analysis on CD3^–^Ter119^–^B220^–^ cells to investigate differences that may not emerge with supervised gating. Among the identified clusters, four showed significant differences between groups. C2 (a CMP subset) was markedly reduced in HFD+EtOH compared to HFD. In contrast, two Ly6G^+^Ly6C^low^ neutrophil subsets, C17 and C15, showed expansion in HFD+EtOH. C17 was increased in HFD+EtOH relative to HFD, while C15, with additional CD48 expression, was significantly upregulated in HFD+EtOH compared to control and HFD groups, indicating expansion of potential granulocyte myeloid-derived suppressor cells (G-MDSCs) (54). Finally, C13, a subset of HSCs, was found only enriched in HFD+EtOH (**Figures 4D, E**; **Supplementary Figure 1L**).

Collectively, these results indicate that HFD+EtOH, following the induction of HSC differentiation into myeloid progenitors, was associated with the expansion of inflammatory Ly6C^high^ and immunosuppressive G-MDSCs, potentially predisposing to dysregulated immune responses.

### Combined alcohol and HFD increased potential osteoclast precursors

**Figure 5:**
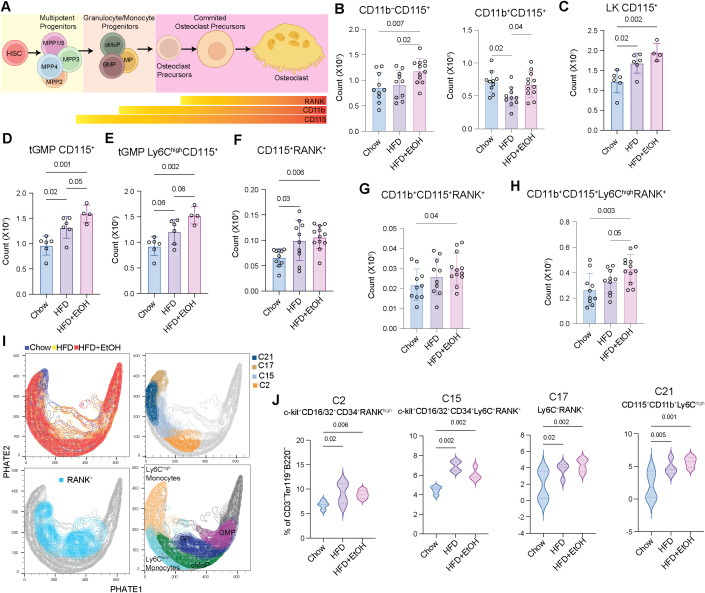
HFD+EtOH enhances potential osteoclast precursors. **(A)** The differentiation trajectory of osteoclast precursors from HSPCs. The number of **(B)** CD11b^–^CD115^+^ and CD11b^+^CD115^+^, **(C)** LK CD115^+^, **(D)** tGMP CD115^+^, and **(E)** tGMP Ly6C^high^CD115^+^ cells, **(F)** CD115^+^RANK^+^, **(G)** CD11b^+^CD115^+^RANK^+^, and **(H)** CD11b^+^CD115^+^Ly6C^high^RANK^+^ cells. **(I)** PHATE plots illustrating the distribution of groups and identified clusters (top), overlapped with the RANK^+^, GMP, GP, and monocytes population derived from supervised analysis (bottom). **(J)** The frequency of identified clusters.

Since enhanced monopoiesis increases the number of potential osteoclast precursors (30), we next examined the expression of CD115 and RANK by flow cytometry (**Figure 1C**) to assess the frequency of potential and committed precursors (55) (**Figure 5A**). Compared to HFD and chow, the HFD+EtOH diet resulted in a significant enhancement in the total CD11b^–^CD115^+^ bone marrow cells. In contrast, the CD11b^+^CD115^+^ population was comparable between HFD+EtOH and chow but markedly reduced in the HFD group relative to both (**Figure 5B**). This indicates that the expansion of CD115^+^ cells by HFD+EtOH may have occurred within the early CD11b^–^ progenitors, potentially in as early as tGMPs, rather than the more mature CD11b^+^ cells. The osteoclastogenesis potential of CD11b^–^ bone marrow cells was shown to be higher than that of CD11b^+^ cells (56).

We next assessed the expression of CD115 across the progenitors’ trajectory of differentiation toward monocytes. Both HFD and HFD+EtOH groups showed a higher number of LK CD115^+^ and tGMP CD115^+^ cells compared to chow, with tGMP CD115^+^ cells further increased in HFD+EtOH relative to HFD (**Figures 5C, D**). Dividing tGMPs into Ly6C^high^ and Ly6C^low^ populations (**Figure 1C**), revealed that Ly6C^high^CD115^+^ tGMPs were significantly enriched in HFD+EtOH compared to chow, with only modest increases relative to HFD (**Figure 5E**), while no differences were detected in Ly6C^low^CD115^+^ tGMPs between the groups (**Supplementary Figure 2A**).

We next examined RANK expression as a marker of committed osteoclast precursors. Total CD115^+^RANK^+^ bone marrow cells were significantly increased in both HFD and HFD+EtOH relative to chow (**Figure 5F**). Similarly, CD11b^+^CD115^+^RANK^+^ cells were also increased in HFD+EtOH compared to chow (**Figure 5G**). In contrast, CD11b^–^CD115^+^RANK^+^ cells showed no significant differences across groups (**Supplementary Figure 2B**), consistent with the expected acquisition of RANK expression in more mature osteoclast precursors. Together with our earlier observation, these findings suggest that HFD+EtOH primarily expands potential immature osteoclast precursors (CD11b^–^CD115^+^), while committed precursors (CD115^+^RANK^+^) are enriched within the CD11b^+^ fraction under both HFD and HFD+EtOH conditions.

Ly6C^high^ precursors are more osteoclastogenic than Ly6C^low^ cells (57, 58). Stratification by Ly6C revealed that CD11b^+^CD115^+^Ly6C^high^RANK^+^ cells were significantly elevated in HFD+EtOH compared to both chow and HFD (**Figure 5H**); while CD11b^+^CD115^+^Ly6C^low^RANK^+^ cells were unchanged across groups (**Supplementary Figure 2C**). This suggests that HFD+EtOH specifically was consistent with the differentiation of a more committed, fusion-competent osteoclast precursor pool.

We next applied unsupervised clustering with PhenoGraph to identify rare populations of osteoclast precursors. Among identified clusters, C2 (c-kit^+^CD16/32^+^CD34^+^RANK^high^), C15 (c-kit^+^CD34^+^CD16/32^+^Ly6C^+^RANK^+^), C17 (Ly6C^+^ RANK^+^), and C21 (CD115^+^Ly6C^high^CD11b^+^) were enriched in both HFD and HFD+EtOH compared to chow. Projection of clusters and supervised gated populations onto the PHATE graph revealed that C2 corresponded to a cMoP population, C17 and C21 are Ly6C^high^ monocytes, and C15 is an intermediate population between cMoP and Ly6C^high^ monocytes (**Figures 5I, J**; **Supplementary Figure 2D**). It was shown that incubating RANKL with purified cMoP produces osteoclasts, suggesting that RANK might be expressed in osteoclast precursors as early as cMoP (59).

Furthermore, some clusters were significantly downregulated in both HFD and HFD+EtOH compared to chow, including C1 (CD16/32^+^CD34^+^), C3 (CD115^+^), C4 (CD115^high^CD11b^+^RANK^high^), and C13 (Ly6C^high^CD11b^high^). With the exception of C13, other clusters overlapped with Ly6C^low^ monocytes (**Supplementary Figures 2E, F**). Further, C12 (RANK^+^) and C19 (c-Kit^+^RANK^+^) were enriched, and C16 (CD16/32^+^CD34^+^CD115^high^Ly6C^+^CD11b^+^RANK^high^) was depleted in HFD compared to chow (**Supplementary Figures 2E, G**).

Collectively, these findings suggest that both HFD and HFD+EtOH expand CD115^+^ osteoclast precursors and committed RANK^+^ precursors, with a significantly greater effect observed in the HFD+EtOH group compared to HFD alone. This expansion was consistent with an increase in osteoclast-competent Ly6C^high^ precursor populations.

### Combined alcohol and HFD increases the osteoclastogenic potential of HSPCs

**Figure 6:**
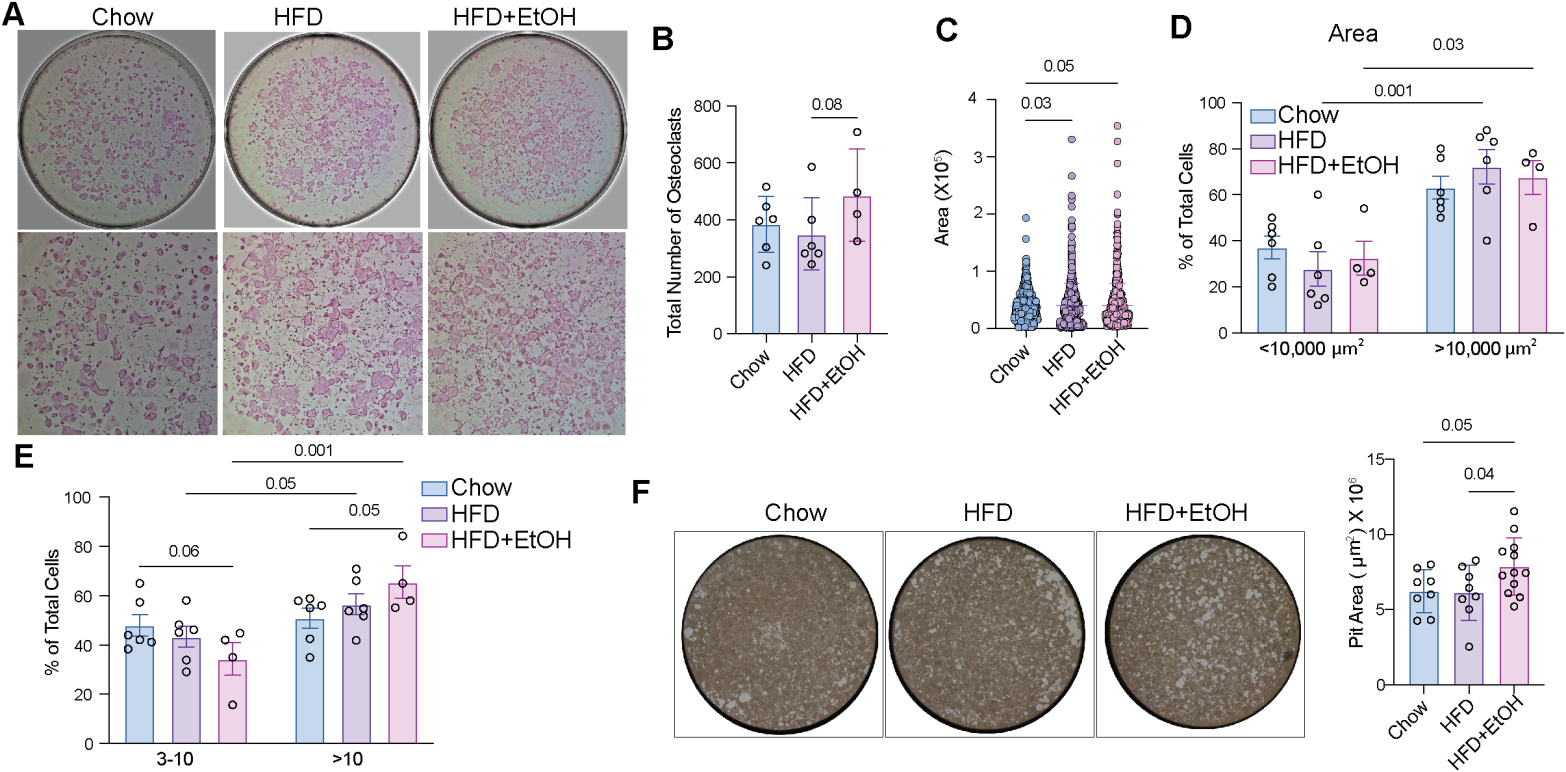
Combination of alcohol and HFD enhances bone marrow cells’ ability to differentiate into osteoclasts. **A)** Bone marrow cells differentiated to osteoclasts and stained with TRAP. **B)** The total number of osteoclasts, defined as TRAP^+^ cells with >3 nuclei. **C)** The area of osteoclasts. Each dot represents one osteoclast**. D)** The frequency of osteoclasts stratified by size within each group. **E)** The frequency of the cells having 3–10 or more than 10 nuclei within each group. **F)** Representing differentiated osteoclasts on a calcium-coated surface (left) and the Pit area was calculated using ImageJ (right).

We observed that combining HFD and alcohol increased the pool of potential osteoclast precursors. To evaluate the osteoclastogenic capacity of these cells, bone marrow cells were stimulated with RANKL and M-CSF *in vitro*, followed by TRAP staining to identify osteoclasts, and culturing on calcium-coated plates to assess resorptive activity (**Supplementary Figure 3A**). The total number of osteoclasts, defined as TRAP-positive cells containing three or more nuclei, showed a modest increase in HFD+EtOH compared to HFD (**Figures 6A, B**). However, the mean osteoclast area in HFD and HFD+EtOH was significantly greater than in chow (**Figure 6C**). We then stratified osteoclasts based on size. Both the HFD and HFD+EtOH groups exhibited a significant shift toward a higher proportion of giant osteoclasts (>10,000 μm²) (**Figure 6D**). This indicates that the fusion capacity of precursors increased, leading to the formation of bigger cells. To that end, we compared the area of all osteoclasts. Since enhanced fusion of pre-osteoclasts is correlated with their multinucleation, we compared the number of nuclei per cell. For both HFD and HFD+EtOH, as in the osteoclast area comparison, we observed a within-group shift toward a greater number of cells with more than 10 nuclei (**Figure 6E**). The Pit assay showed that osteoclasts from HFD+EtOH mice exhibited a significantly larger resorbed area compared to those from HFD and chow (**Figure 6F**). Together, these findings indicate that HFD+EtOH was associated with an increased in the pool of osteoclast precursors and potentially their fusion capacity, leading to the formation of larger multinucleated osteoclasts with heightened resorptive activity.

### Alcohol and HFD impair bone modeling

**Figure 7:**
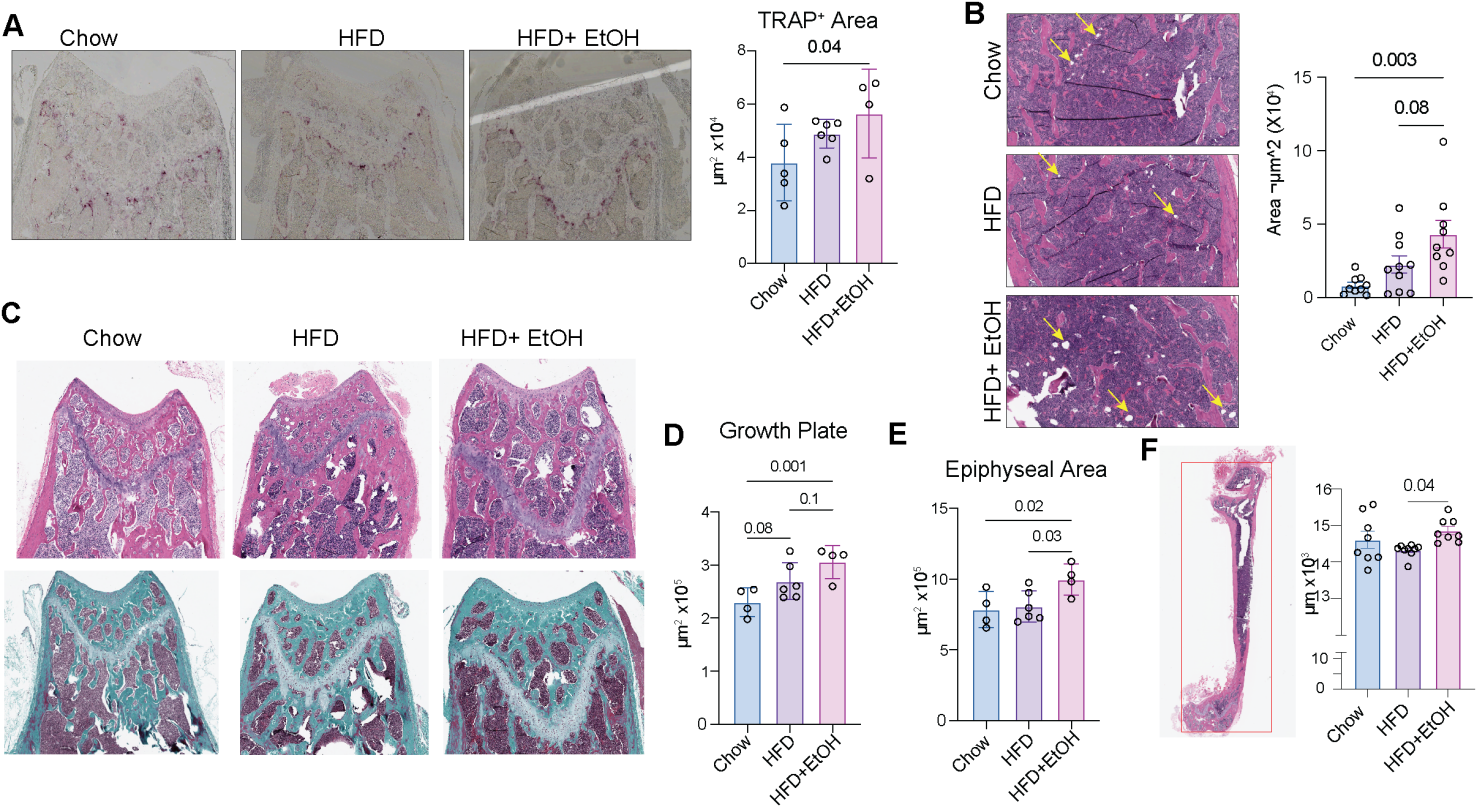
Altered bone remodeling following combined alcohol and HFD. **A**) TRAP-stained decalcified bone sections and measurement of the TRAP^+^ area at the distal femur. **B**) Lipid accumulation in H&E-stained bone sections was manually identified and quantified using QuPath. **C**) H&E (top) and trichrome (bottom) staining of bone sections. **D**) The growth plate and **E**) epiphyseal area were measured using H&E-stained sections. **F**) The H&E-stained femur was used to measure the length of the bone.

We next assessed whether the increased frequency of osteoclast precursors and their resorptive activity were associated with changes in bone. Decalcified femur sections were stained with TRAP, and the TRAP-positive area was quantified across equivalent distal regions using pixel classification (**Supplementary Figure 3B**). Consistent with *in vitro* observation indicating enhanced potential osteoclasts precursors, it was observed that the TRAP^+^ area was significantly greater in HFD+EtOH compared to chow (**Figure 7A**). Because adipocytes establish a pro-osteoclastogenic bone-marrow environment (60), bone marrow lipid content in H&E–stained femur sections were evaluated for lipid accumulation, identified as clear circular droplets within the marrow compartment. HFD+EtOH displayed a marked lipid accumulation increase relative to chow, with a modest increase compared to the HFD (**Figure 7B****).**

To evaluate whether the presence of active osteoclasts impacted bone growth, we analyzed growth plate, epiphyseal area and height in H&E-stained bone sections (**Figure 7C**; **Supplementary Figure 3C**). The growth plate area was significantly larger in the HFD+EtOH group compared to chow, with HFD alone showing only a modest increase (**Figure 7D****).** In line with this, epiphyseal area was markedly greater in HFD+EtOH relative to both chow and HFD groups (**Figure 7E****).** Similar differences were also observed in epiphyseal height (**Supplementary Figure 3D**). To assess the impact of these changes on longitudinal bone growth, we measured femur length and observed that it was significantly greater in HFD+EtOH group than in HFD, but not different from chow (**Figure 7F****).**

Together, these observations suggest that while the HFD+EtOH diet produced substantial marrow lipid accumulation and markedly increased TRAP-positive resorptive area in the distal femur, it paradoxically enhanced growth plate area and epiphyseal area relative to chow and HFD, resulting in enhanced femur length compared to HFD but comparable to chow. These findings indicate that HFD+EtOH, in addition to affecting the differentiation of HSPCs toward osteoclast precursors, may also delay chondrocyte hypertrophy or prolong the proliferative activity of the growth plate. This results in a longer period of cartilage production and epiphyseal expansion, leading to increased epiphyseal height and, compared with HFD alone, enhanced longitudinal growth.

## Discussion

Diets rich in alcohol or fat may cause excessive or inappropriate immune activation, immune deficits, and further promote organ damage beyond liver injury (61, 62). In MASLD, multiple immune cells release inflammatory mediators, contributing to the establishment of a pro-inflammatory environment (4, 23, 63). Alcohol consumption can further exacerbate this inflammatory state through multiple mechanisms, including increased gut permeability and subsequent endotoxin translocation (23, 64). The chronic inflammatory milieu established in MASLD and MetALD may disrupt normal bone marrow hematopoiesis, thereby impairing immune competence (65) and exacerbating liver pathology (11, 66). Although numerous studies have investigated the effects of a high-fat diet on the immune system and hematopoiesis, few have examined the combined impact of a high-fat diet and alcohol consumption (67). It is expected that combining these diets could synergistically impact immune cells, yet explicit models integrating both insults remain scarce (68, 69). Given that many individuals with liver disease exhibit both alcohol use and metabolic syndrome, we established two complementary experimental models to better represent these common clinical comorbidities as well as delineate the exacerbating impact of alcohol on high-fat diet-driven pathology. While liver pathology metrics were not included in the present manuscript, similar models, with variations in diet composition or duration, have shown significant hepatic injury and recapitulate key features of MASLD and MetALD (69).

Our findings revealed an overall enhanced LK and LSK populations, suggesting that HSC differentiation is biased toward lineage-negative progenitor cells in both HFD and HFD+EtOH. Further, HFD alone tends to reduce the HSC pool, whereas HFD+EtOH expands it. These observations suggest that in HFD+EtOH, HSCs maintain their self-renewal capacity despite ongoing differentiation pressure, whereas in HFD alone, diminished self-renewal likely contributes to the observed reduction in the HSC pool. Indeed, downregulation of c-kit, which identifies HSCs with enhanced self-renewal and long-term engraftment potential (39, 40), was observed only in HFD+EtOH. Consistent with our results, prior studies reported that HFD decreases the frequency of HSCs (CD150^+^CD34^+^) and CMPs (Lin^-^Sca-1^-^c-Kit^+^CD34^+^CD16/32^low^ among LK), while increasing multipotent myeloid and monocytes (70). This was in accordance with the decreased proliferation of HSC *in vitro*, characterized by higher G0/G1 and lower M-phase populations, and a decrease in their multilineage reconstitution potential following transplantation to chow diet mice. These observations suggest that although HSPCs in an obesogenic environment exhibit enhanced differentiation, they may concurrently undergo functional exhaustion (70). In contrast, another study showed that the diet effects are time-dependent, with short-term HFD (1–4 weeks) reducing LT-HSCs, GMP, and CMP myeloid progenitors, and prolonged exposure (≥12 weeks) expanding the LT-HSC pool (71). In a third study, HSCs remained quiescent in the HFD mode (72). These inconsistencies could be due to differences in the type of HFD diet, the duration of HFD, the microbiome among animal facilities, the genetic background of the model, the sex of the animals, and the status of the leptin hormone (11, 73). Additionally, variation in whether absolute cell numbers, frequencies within total bone marrow, or frequencies within defined progenitor subsets were reported can affect the interpretation of the results. For example, a study showed that while the number of HSCs decreased with HFD, their frequency increased among the total cells (74).

We observed that at the early multipotent progenitor level, the HFD diet, consistent with HSC depletion, resulted in a reduction in MPP1. At the committed multipotent progenitor level, the HFD+EtOH diet led to an expansion of the myeloid-biased MPP3 subset, potentially promoting a shift toward myeloid lineage differentiation. Despite the differential impact on early and biased multipotent progenitors, a common observation for both diets was a significant reduction in total CMPs, suggesting accelerated consumption of these cells toward myeloid lineage differentiation. This was aligned with a modest increase in MP+cMOPs under HFD, and a further enhancement of granulocyte-monocyte progenitors (MP+cMOP, GMP, and GP) by the HFD+EtOH diet. Enhanced expression of Ly6C on MDPs and MP+cMOPs in both HFD and HFD+EtOH indicates a commitment toward the monocyte lineage and an increased potential for pro-inflammatory function (52, 53). This was further supported by an increase in the absolute number of monocytes, particularly Ly6C^high^ cells in the HFD+EtOH group. Ly6C^high^ monocytes produce pro-inflammatory cytokines and differentiate into inflammatory macrophages or monocyte-derived dendritic cells (52, 53). Enhanced Ly6G^+^Ly6C^low^ G-MDSCs were also observed in the HFD+EtOH group. It was reported that alcohol consumption may enhance the differentiation of myeloid progenitors to increase G-MDSCs (54).

We observed that while HFD alone induced only a modest accumulation of marrow lipids, the addition of alcohol produced a striking increase in adipocyte content. Although bone marrow adipose tissue provides essential nutrients for HSCs, its expansion can instead exert inhibitory effects on normal hematopoietic function (11, 75). Expansion of bone marrow adiposity observed in chronic metabolic diseases (76) is a contributing factor in enhanced myelopoiesis. Increased bone marrow fat content and associated changes in adipokine/cytokine profiles increasingly suppress lymphoid differentiation and support myeloid expansion, potentially contributing to the systemic inflammation and immune dysregulation observed in liver disease (60, 76–80). Adipose-derived S100A8/A9 has been shown to activate TLR4/MyD88 signaling in adipose tissue macrophages, resulting in the release of IL-1β, which then binds to the IL-1 receptor on bone marrow myeloid progenitors to promote the production of monocytes and neutrophils (73, 81).

We have previously shown that enhanced monopoiesis and an increased number of potential osteoclast precursors in a non-human primate model of chronic alcohol consumption (30). MASLD was reported to cause loss of skeletal muscle mass and function, affecting up to 60% of those with end-stage liver disease (15, 16), decreased BMD, and increased risk of bone fracture (17, 82) due to upregulation of inflammatory mediators such as iNOS, NLRP3, and stress-response genes (83). Alcohol consumption has also been extensively reported to disrupt bone remodeling and increase the risk of bone fractures (84–86) by affecting mesenchymal stem cells (MSCs) and their differentiation into osteoblasts (87, 88), as well as HSPCs and the osteoclast lineage (89, 90). Accordingly, we observed that the HFD+EtOH diet increased the frequency of CD11b^–^CD115^+^ cells and Ly6C^high^CD115^+^ tGMPs. CD11b^–^ bone marrow cells exhibit a greater potential for osteoclastogenesis compared to CD11b^+^ cells (56). These findings suggest that alcohol-driven chronic inflammation maintains an expanded pool of CD115^+^ cells, thereby enlarging the reservoir of osteoclast precursors poised for differentiation (91–93). We also observed a significant expansion of committed osteoclast precursors (CD115^+^RANK^+^) in both experimental groups, with an additional increase in the CD11b^+^CD115^+^Ly6C^high^RANK^+^ subsets under HFD+EtOH. Together, these results indicate that HFD+EtOH predominantly expands osteoclast precursors (CD11b^–^CD115^+^). Enrichment of committed precursors (CD115^+^RANK^+^) occurred under both HFD and HFD+EtOH conditions, with a significantly greater effect observed in the HFD+EtOH group compared to HFD alone, through expression of RANK in Ly6C^high^ cells. Ly6C^high^ cells are more likely to develop into osteoclasts than Ly6C^low^ cells (57, 58).

Assessment of the osteoclastogenesis capacity of bone marrow cells showed that the total number of osteoclasts modestly increased in HFD+EtOH compared to HFD. Importantly, HFD+EtOH mice showed the greatest increase in multinucleation, with a substantial fraction of osteoclasts containing more than 10 nuclei, which correlated with elevated bone resorptive function. Multinucleation is a hallmark of osteoclast functional maturation and directly correlates with resorption efficiency (94). Consistently, TRAP staining of femoral sections revealed a significant increase in TRAP^+^ area in HFD+EtOH compared with chow. These findings indicate that the combined dietary stress not only expands the pool of osteoclast precursors but may also be associated with enhanced fusion, resulting in giant, hyperactive osteoclasts.

These observations were accompanied by alterations in growth plate morphology. Both growth plate area and epiphyseal thickness were substantially greater in HFD+EtOH compared with chow, with HFD alone showing only modest effects. Despite these changes, total femur length was not significantly different between chow and HFD+EtOH groups, though it was greater than in HFD alone. Importantly, considering animals’ age, their skeletal growth potentially is active. Therefore, our findings may reflect disruption of developmental growth processes. In addition, these observations suggest that metabolic and inflammatory stressors not only skewed hematopoiesis and enhanced osteoclastogenesis but also independently influenced chondrocyte proliferation and/or hypertrophy within the epiphyseal region, highlighting distinct regulatory mechanisms governing osteoclast activity and growth plate dynamics.

The growth plate contains three principal zones: germinal, proliferative, and hypertrophic. Under normal conditions, proliferative chondrocytes divide, form columnar stacks, and then exit the cell cycle to undergo hypertrophic enlargement. Enlargement of the growth plate reflects a disruption in the timing of chondrocyte maturation. It may signal either 1) delayed hypertrophy due to sustained proliferative signaling or 2) prolonged or excessive proliferative activity within the proliferative zone that outpaces terminal differentiation and ossification (95–97). The transition between proliferation and hypertrophy is chiefly regulated by a negative feedback loop between parathyroid hormone-related protein (PTHrP) and Indian hedgehog (Ihh) (98). If hypertrophic differentiation is delayed, hypertrophic zone expansion is restrained while proliferative columns persist longer. This can increase overall growth plate thickness because proliferative chondrocytes accumulate without efficiently transitioning into hypertrophic chondrocytes. Elevated PTHrP signaling, excess growth hormone, IGF-1, or FGFR3 inhibition maintains chondrocytes in a proliferative state and delays hypertrophy. Increased PTHrP signaling has been reported under high-fat diet conditions (99–102), while the effects of alcohol consumption appear dependent on exposure duration and dose (103, 104). Alternatively, growth plate enlargement may result from extended proliferation rather than delayed hypertrophy. Studies show that when proliferation continues further down the chondrocyte columns, the growth plate appears larger, and bone elongation persists longer. Chronic stimulation of proliferating chondrocytes or reduced apoptotic clearance of hypertrophic cells can thus broaden the plate (105). Overall, our findings suggest that combined metabolic and ethanol stress may alter developmental cartilage dynamics, warranting future studies to distinguish delayed hypertrophy from prolonged proliferation through targeted molecular analyses such as examining markers of hypertrophic differentiation (e.g., Col10a1, Mmp13) and proliferation (e.g., Ki67, PCNA).

Together, this study shows that HFD in combination with alcohol affects hematopoiesis, promoting myelopoiesis and osteoclastogenesis, which further impacts bone modeling, but it is not without limitations. We only used male mice because it was reported that HFD-mediated myelopoiesis is significantly more pronounced in male mice compared to female mice (106). However, the reported sex-specific differences in hematopoiesis (107), bone loss (17, 108), and responses to both HFD (106, 109) and alcohol (110) indicate the need to include female mice in future studies to determine the extent to which these findings generalize across sexes. In addition, HSC subsets were analyzed in a limited sample size, and blood counts of monocytes, neutrophils, or DCs were not determined. Future studies will include peripheral blood phenotyping to determine whether signatures identified in bone marrow are reflected systemically, which could ultimately serve as predictors of skeletal complications in patients with MASLD and MetALD. Although we observed differential phenotypes among HSCs under both diets, we have not assessed HSCs’ functional competence, including quiescence and exhaustion. Future research should also employ adoptive bone marrow transfer and lineage tracing to evaluate the repopulating capacity of HSCs after each diet. In addition, since this study did not include direct quantitative assessments of bone mass and its microarchitecture using specialized techniques such as µCT or densitometric analyses, conclusions regarding bone modeling remain speculative. Further assessment of bone microarchitecture and length, lipid buildup, and chondrocyte activity should be conducted. In addition, future studies should investigate the molecular mediators linking marrow adiposity, growth plate expansion, and osteoclast activation, with particular attention to inflammatory cytokines, lipid metabolites, and endocrine factors. Together, further characterization of bone microarchitecture and HSC function, along with assessment of blood cells and markers in association with liver pathology metrics, may help identify predictors of skeletal complications and reveal therapeutic strategies to mitigate skeletal fragility in populations at risk due to a high-fat diet and alcohol exposure.

## Data Availability

The original contributions presented in the study are included in the article/**Supplementary Material**. Further inquiries can be directed to the corresponding authors.
