# Supplementary material for: Alcohol consumption exacerbates high-fat diet-mediated disruptions in myelopoiesis and osteoclastogenesis in mouse models of metabolic dysfunction-associated liver diseases
Source: Front Endocrinol (Lausanne). 2026 Mar 20;17:1783132. doi: 10.3389/fendo.2026.1783132 (PMC13046482; doi:10.3389/fendo.2026.1783132)
Supplement: Supplementary file 1 [file Image1.pdf]

Supplementary Figures

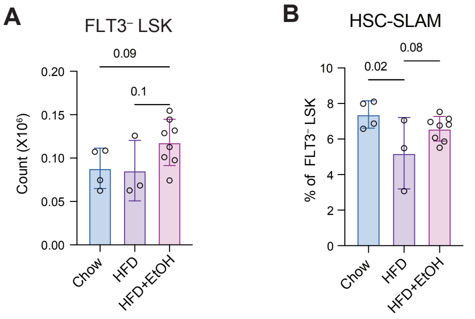

C

		HFD vs Chow	HFD+EtOH vs Chow	HFD+EtOH vs HFD
HSC-SLAM	CD150	<0.001	0.0002	0.0476
	CD34	0.0027	0.0016	
	CD48	0.0239		
	Sca-1	0.0174		
	c-Kit		0.0048	
LT-HSC	CD150	0.0020	0.0033	
	CD34			
	CD48	0.0106		
	Sca-1			
	c-Kit	0.0038	0.0051	

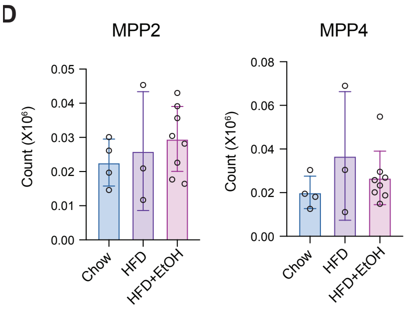

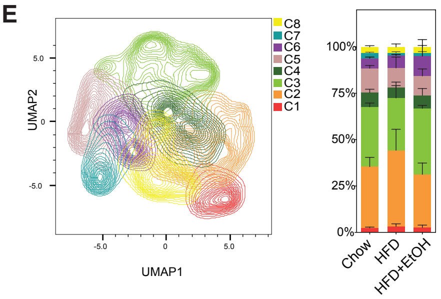

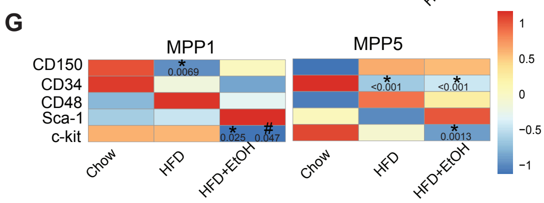

F

		HFD vs Chow	HFD+EtOH vs Chow	HFD+EtOH vs HFD
MPP2	CD150			
	CD34	0.0198	0.0316	
	CD48	0.0090	0.0115	
	Sca-1		0.0432	
	c-Kit			
MPP3	CD150			
	CD34	<0.001	<0.001	
	CD48			
	Sca-1			
	c-Kit		0.0017	
MPP4	CD150			
	CD34			
	CD48			
	Sca-1			
	c-Kit	0.0230		

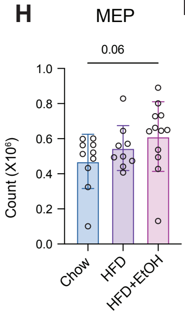

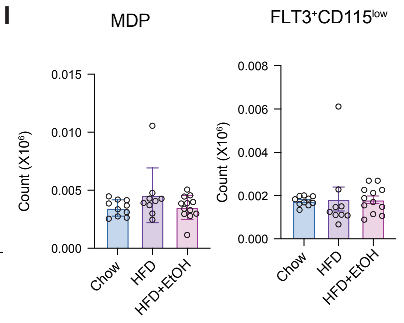

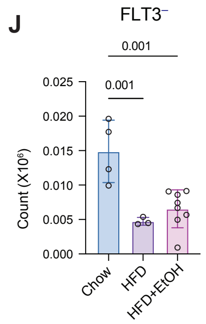

K

		HFD vs Chow	HFD+EtOH vs Chow	HFD+EtOH vs HFD
MDP	CD11b	<0.001	<0.001	
	CD16/32	0.001	0.001	
	CD34			
	CD115			
	Ly6C	0.0382	0.0020	
	c-kit	<0.001	<0.001	
GMP	CD11b	<0.001	<0.001	<0.001
	CD16/32	0.0208		0.0014
	CD34	<0.001	<0.001	<0.001
	CD115			
	Ly6C			
	c-kit	<0.001	<0.001	<0.001
MP+cMOP	CD11b	<0.001	<0.001	
	CD16/32			
	CD34			
	CD115	0.001		0.0008
	Ly6C	<0.001	<0.001	0.0085
	c-kit	<0.001	<0.001	
GP	CD11b	<0.001	<0.001	
	CD16/32	0.0083		
	CD34	<0.001	<0.001	
	CD115	0.0002	<0.001	
	Ly6C			
	c-kit	<0.001	0.0093	

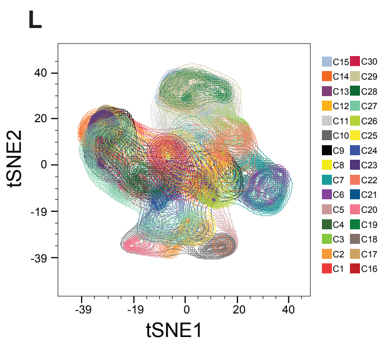

Supp. Figure 1- Differential impact of HFD and HFD+EtOH on hematopoietic stem cells. **A**) Number of FLT3⁻ LSK among the groups. **B**) The ratio of HSC-SLAM among FLT3⁻ LSKs. **C**) P-values of comparisons presented in Fig. 1H. Cell count of **D**) MPP2 and MPP4 among total cells. **E**) UMAP plot illustrating clusters identified by FlowSOM. **F**) P-values of comparisons presented in Fig. 2F. **G**) MFI of key hematopoietic markers on the surface of early multipotent progenitors MPP1 and MPP5. The number of **H)** MEP, **I)** MDP, FLT3^{+}CD115^{low,} and **J)** FLT3⁻ cells. **K**) P-values of comparisons presented in Fig. 3I. **L)** t-SNE plots illustrating identified clusters.

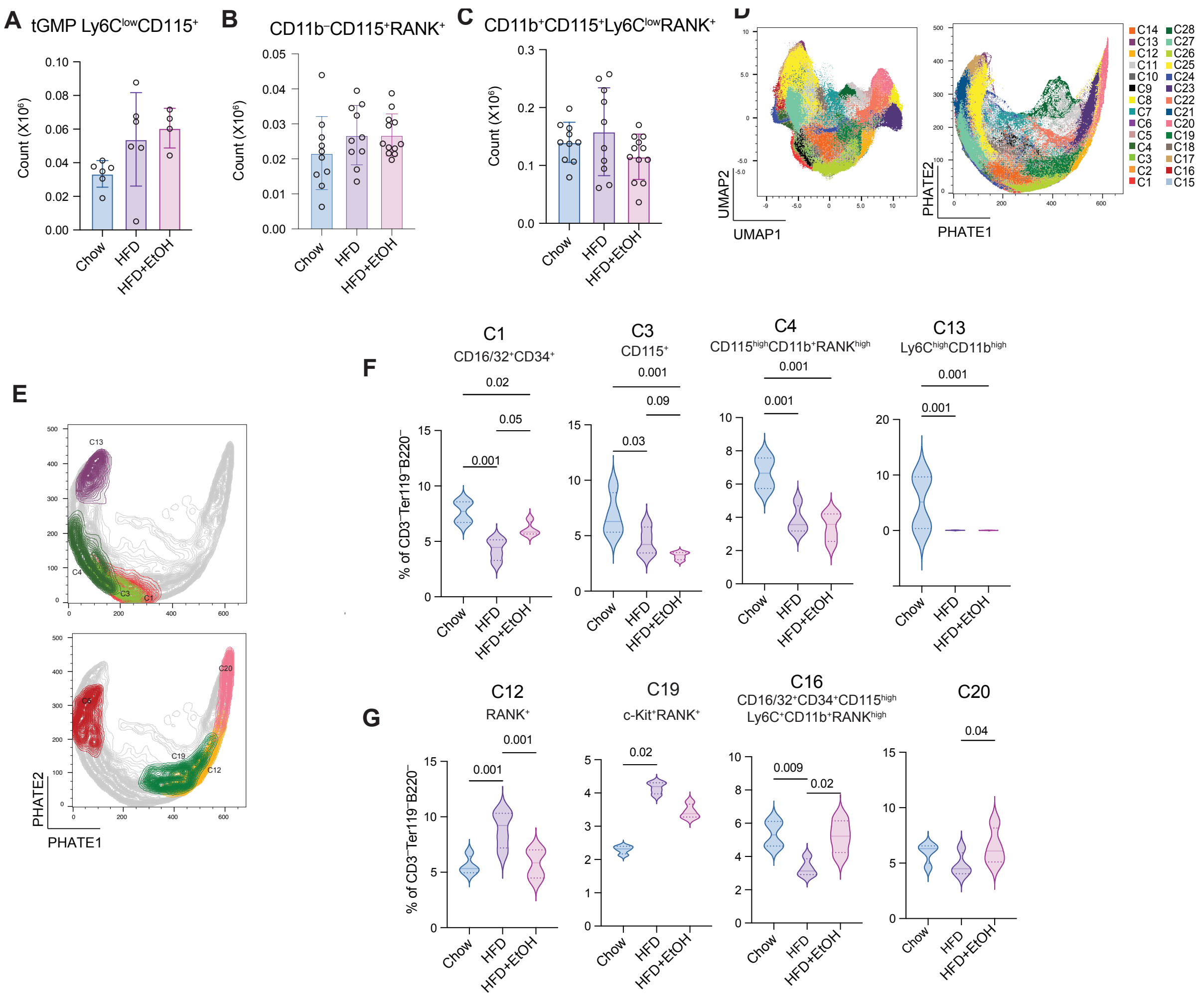

Supp. Figure 2- Alcohol and HFD enhance potential osteoclast precursors. The number of **A**) tGMP Ly6C^{low}CD115^{+}, **B**) CD11b^{-}CD115^{+}RANK^{+}, and **C**) CD11b^{+}CD115^{+}Ly6C^{low}RANK^{+} cells. **D**) UMAP and PHATE plots illustrating the distribution of groups and identified clusters. **E)** C1, C3, C4, and C13 (top) and C6, C12, C19 and C20 were overlapped on PHATE plot. **F-G**) The frequency of identified clusters.

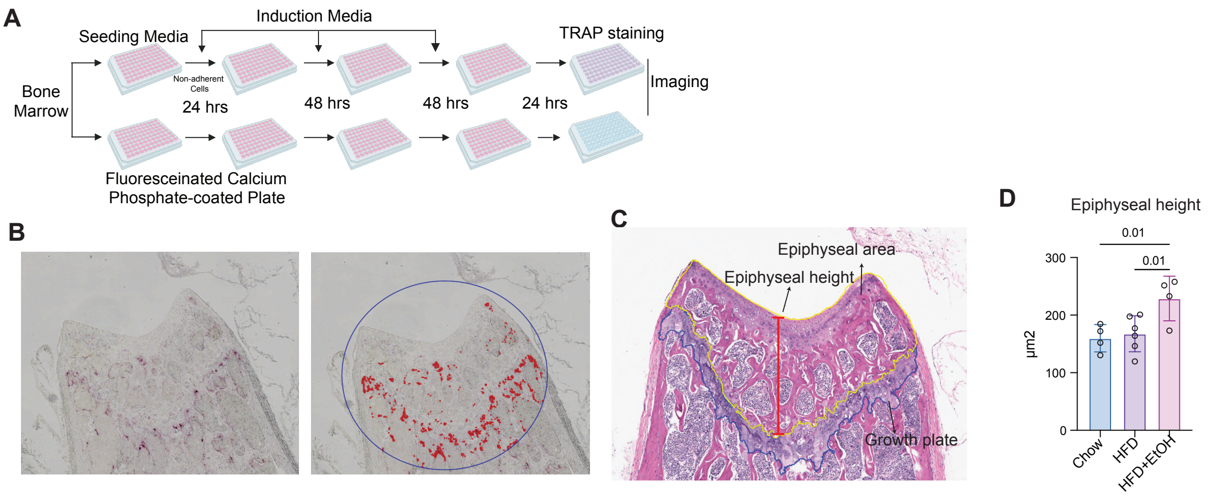

Supp. Figure 3- HFD+EtOH leads to skeletal complications. A) study design to assess the osteoclastogenic potential of bone marrow cells and their resorptive activity *in vitro*. **B**) TRAP-stained decalcified bone sections were used to measure the TRAP^{+} area. A corresponding section of the femur was marked to determine the TRAP^{+} area. **C**) The H&E-stained bone sections were used to measure the area of bone cartilage (marked by blue) and the epiphyseal area (marked by yellow). **D**) The epiphyseal height differences among the groups.
